# Linking in domain-swapped protein dimers

**DOI:** 10.1038/srep33872

**Published:** 2016-09-23

**Authors:** Marco Baiesi, Enzo Orlandini, Antonio Trovato, Flavio Seno

**Affiliations:** 1 Department of Physics and Astronomy, University of Padova, Padova, Italy; 2 INFN-Sezione di Padova, Padova, Italy; 3 CNISM-Unità di Padova, Padova, Italy

## Abstract

The presence of knots has been observed in a small fraction of single-domain proteins and related to their thermodynamic and kinetic properties. The exchanging of identical structural elements, typical of domain-swapped proteins, makes such dimers suitable candidates to validate the possibility that mutual entanglement between chains may play a similar role for protein complexes. We suggest that such entanglement is captured by the linking number. This represents, for two closed curves, the number of times that each curve winds around the other. We show that closing the curves is not necessary, as a novel parameter *G*′, termed Gaussian entanglement, is strongly correlated with the linking number. Based on 110 non redundant domain-swapped dimers, our analysis evidences a high fraction of chains with a significant intertwining, that is with |*G′*| > 1. We report that Nature promotes configurations with negative mutual entanglement and surprisingly, it seems to suppress intertwining in long protein dimers. Supported by numerical simulations of dimer dissociation, our results provide a novel topology-based classification of protein-swapped dimers together with some preliminary evidence of its impact on their physical and biological properties.

In biological systems, proteins rarely act as isolated monomers and association to dimers or higher oligomers is a commonly observed phenomenon^1^,^2^,^3^,^4^,^5^,^6^,^7^,^8^. Recent structural and biophysical studies show that protein dimerization or oligomerization is a key factor in the regulation of proteins such as enzymes^9^, ion channels^10^, receptors and transcription factors^11^,^12^. In addition, this mechanism can help to minimize genome size, while preserving the advantages of modular complex formation^3^. Oligomerization, however, can also have deleterious consequences when non-native oligomers, associated with pathogenic states, are generated^13^,^14^,^15^,^16^,^17^. Specific protein dimerization is integral to biological function, structure and control, and must be under substantial selection pressure to be maintained with such frequency in living organisms.

Protein-protein interactions may occur between identical or non-identical chains (homo or hetero-oligomers) and the association can be permanent or transient^18^. Protein complexes can widely differ based on their affinity. Binding affinities, evaluated for dimers as dissociation constants, can cover up to nine orders of magnitude, highlighting the fact that a strong modulation is necessary to hold up the full protein interaction network^19^,^20^.

The mechanisms for the evolution of oligomeric interfaces and those for the assembly of oligomers during protein synthesis or refolding remain unclear. Different paradigms have been proposed for the evolution of protein oligomers, among which figures three-dimensional (3D) domain swapping^21^,^22^,^23^,^24^,^25^.

Three-dimensional domain swapping is a mechanism through which two or more protein molecules form a dimer or higher oligomer by exchanging an identical structural element (see Figs 1 and 2). Several native (natural/physiological) intra-molecular interactions within the monomeric structures are replaced by inter-molecular interactions of protein structures in swapped oligomeric conformations^26^. Critical in this process is the hinge region, the only polypeptide segment that needs to adopt different conformations in the monomer and in the domain-swapped oligomer. Domain swapping is typically the response of the protein to relieve conformational stress that is present in this hinge region of the monomer. Structures in swapped conformations were reported to perform a variety of functions, and proteins involved in deposition diseases (like neurodegenerative diseases, amyloidosis and Alzheimers disease) have been reported in 3D domain-swapped conformations^27^,^28^,^29^,^30^.

Domain-swapped proteins may assume rather complicated spatial structures, since the swapping arms in their rotation can wind up forming tightly compenetrated structures. Examples are shown in Figs 1 and 2. For instance, the Staphylococcus aureus thioredoxin (Trx) fold mutant (pdb code 3DIE) represented in Fig. 2 clearly illustrates the deep clinging between the two proteins.

The apparent intertwining between proteins assembled in complexes is certainly a distinguishing characteristic of these systems. An interesting issue to explore is the possibility of introducing topology-based descriptors that can capture the entanglement in a robust and measurable way, and that can be related to either some physico/chemical properties or biological functionalities.

For instance, for single chain globular proteins it has been observed that the backbones may entangle themselves into a physical knot^31^,^32^,^33^,^34^,^35^,^36^ that does not disentangle even in the denatured unfolded state^37^. Knotted proteins are interesting because they are rare, and their folding mechanisms and function are not well understood, although it has been proposed that they might increase thermal and kinetic stability^38^,^39^,^40^.

Although knots are mathematically defined only for closed loops^41^, in the last decade there have been several attempts to introduce sufficiently robust and topologically inspired measures of knots in open chains^42^. For a single protein, for instance, one can close its backbone by connecting the N and C termini to a point (chosen randomly) on the surface of a sphere that contains the chain. This sphere can be either very large compared to the chain size (closure at infinity) or it can be replaced by the convex hull of the chain. In all cases the artificial closure can introduce additional entanglement and several ways have been suggested to either mitigate or control this problem^43^,^44^,^45^,^46^.

For two proteins forming a dimer, if one is interested in measuring the degree of mutual entanglement, the notion of a knotted open chain must be generalised to that of a link between two open chains. In analogy with the procedure used for knots, one can think of closing artificially the two backbones to generate two loops. This can be done by joining the ends of each protein at infinity and computing a link invariant, such as the linking number *G_n_*, an integer index that quantifies the number of times that a curve winds around the other (technically, with Gauss integrals evaluated over the protein backbone, *G_n_* detects the degree of homological linking between two closed curves^41^ and can be used to classify proteins^47^).

Two curves are not linked if *G_n_ *= 0 while *G_n_ *= 1 or *G_n_ *= −1 denotes the simplest link between two loops. Since the sign of *G_n_* depends on the orientation of the two curves, in our implementation we choose to follow the standard N-C orientation along the backbones of the proteins. As in knots, the random closure may introduce additional linking between the two chains and an estimate of *G_n_* is necessarily a probabilistic one that requires a sufficiently large number of closure paths^45^.

We denote by *G* the average of the linking number *G_n_* over many closures. Along with *G*, we consider a *Gaussian entanglement* indicator, *G*′, computed with the same method adopted for *G* but without closing the curves. We show that *G*′ strongly correlates with its topological counterpart *G*. Both estimators are used to analyse a non-redundant set of 3D domain swapped dimers. It turns out that several dimers present a high degree of mutual entanglement.

The short CPU time required to estimate *G*′ allows a quick systematic mining of linked dimers from protein databanks. With a computationally much heavier test, for some dimers we check whether this measure of entanglement is robust or is just an artifact of the specific crystallographic structure found in the database. This is done by performing simplified molecular dynamics simulations, starting from several native structures with non-trivial values of *G*′. The time evolution of *G*′(*t*) during the process of dissociation of the dimer reveals additional information on the amount of intertwining of the two proteins.

## Results

### Databank

Within the protein databank we have found *n*_*D*_ = 110 non-redundant domain-swapped proteins and for each of them we have computed the average linking number *G* and the Gaussian entanglement *G*′ for open chains (see Methods for details, and below). The results are reported in Table 1, ranked for increasing *G*′. The table also indicates whether the dimer is human (33 cases, tagged with a “h”) or not, and reports the number *N* of *C*_*α*_ atoms of each protein forming the dimer.

### Protein mutual entanglement estimators: *G* e *G*′

As a first indicator of the mutual entanglement of two proteins belonging to a given dimer, we consider the Gauss integral *G_n_* computed over the pair of loops obtained by closing randomly each protein *C*_*α*_ backbones on a sphere (see Fig. 3 and Methods for details). For a given closure, *G_n_* is an integer^41^ but, once averaged over several random closures, its mean value *G* is eventually a fractional number.

Alternatively, we can apply the use of the Gauss integrals to open backbones. This measure, *G*′, is certainly not a topological invariant anymore, but nevertheless it captures the interwinding between the two strands and does not require averages over many random closures. By computing these two quantities on the whole set of swapped domains in our dataset, we can notice that there is a strong correlation between *G* and *G*′ (see Fig. 4). This result validates, at least for the domain-swapped proteins, the use of *G*′ as a faithful measure of the mutual entanglement. The reason to prefer *G*′ is twofold: First, the estimate of *G*′ does not require a computationally expensive averaging over different closure modes. Second, there are cases in which the closure procedure does not work properly, giving rise to an unreasonable value of *G* (compared to *G*′, see the point with *G *> 2 and *G*′ ≈ 0 in Fig. 4).

### Analysis of the Gaussian entanglement *G*′

The histogram reproducing the number of swapped dimers with a given *G*′ is plotted in Fig. 5(a): The distribution of *G*′ is fairly well fitted by a Gaussian with mean ≈ −0.1 and standard deviation ≈0.63. The plot has high fraction of cases with −1 < *G*′ < 1, suggesting that most of the 3D domain-swapped dimers are not linked. On the other hand, there is a consistent percentage of structures that exhibit a non trivial value of |*G*′|. In particular, in Table 1 there are 15 structures with *G*′ < −1 and 4 dimers have *G*′ > 1. Hence, more than 15% of the dimers in our databank have |*G*′| higher than 1.

The figures also tell us that the statistics of mutual entanglement displays an asymmetry towards more negative values of *G*′. Indeed, in Table 1 one could notice that about two thirds of the dimers have *G*′ < 0. For obtaining a better evaluation of the spread and average value of *G*′, we analysed the empirical cumulative distribution function *F*(*G*′), namely the fraction of configurations that have a value at most equal to *G*′, see solid lines in Fig. 5(b). These have been fitted by an error-function with non-zero average. The fit yields average 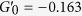 and standard deviation Δ*G*′ = 0.853 (the corresponding fit is shown as a dashed line in Fig. 5(b)). If the fit is restricted to non-human dimers, we get 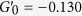 and Δ*G*′ = 0.830, while for the human dimers we get 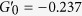 and 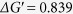. Again, mean values are lower than zero both for the case of human proteins and non-human ones. A fraction of data ≈64% (non-humans) and ≈70% (humans) have *G*′ < 0.

The asymmetry in the distribution in favor of structures with negative *G*′ is slightly more marked for human swapped dimers. This could be explained by the fact that, for human proteins, the interface between the secondary structures of the two monomers is mainly formed by swapped *β*-structures: the preferential right-handed twist of the *β*-sheets together with the higher frequency of anti-parallel pairings may imply a negative value for *G*′.

In order to verify whether our measure of mutual entanglement is affected by some bias that can be introduced by the different lengths of proteins, in Fig. 6 we plot *G*′ as a function of the number of amino acids in the proteins forming the dimers. As a matter of fact we see that the mean value of 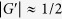 is not varying significantly in the range under investigation, which includes protein lengths ranging from about 50 to 550 amino acids. Only fluctuations are larger at small length due to the presence of more data. Therefore we conclude that, in our dataset, the Gaussian entanglement is a parameter intrinsic to the dimers and it is not affected by entropic effects induced by the length of the protein.

### Dynamical entanglement

**Figure 1:**
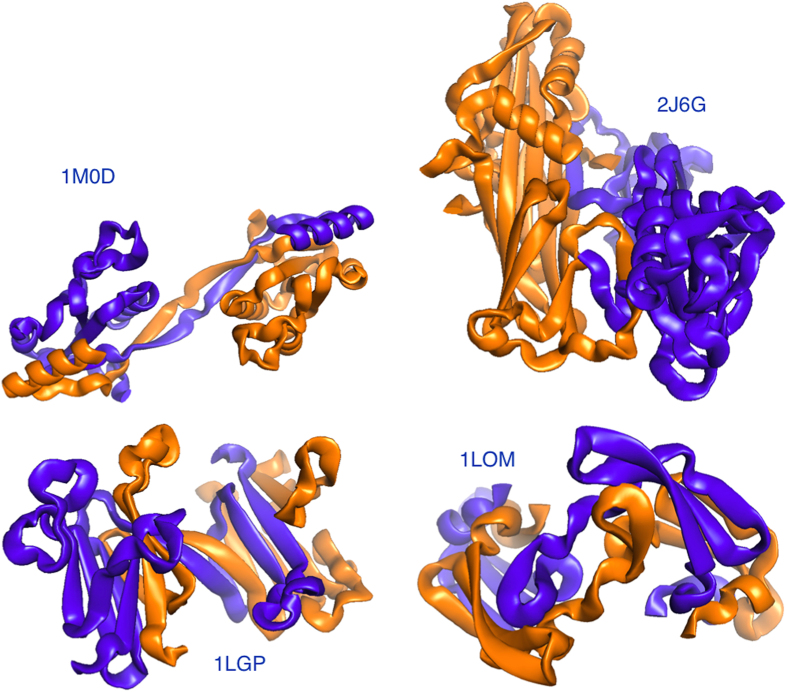
Some domain-swapped dimers with high, negative linking number.

**Figure 2:**
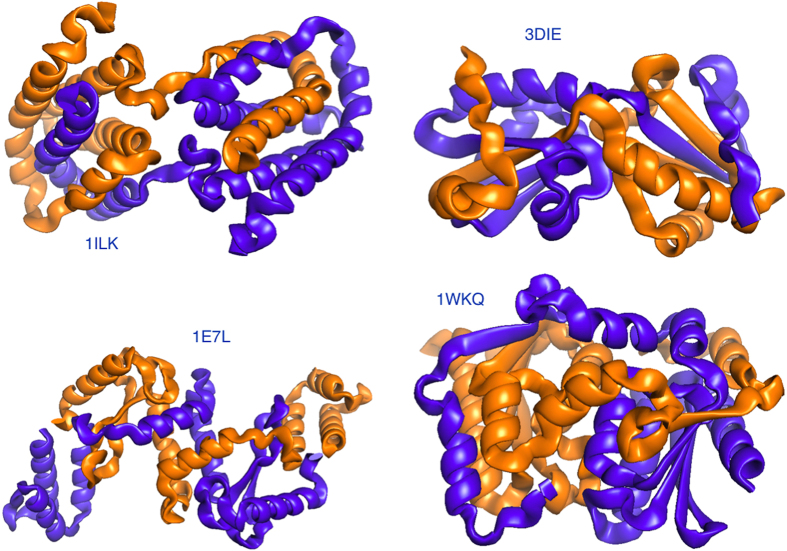
Some domain-swapped dimers with high, positive linking number.

The values of *G*′ are easy to compute from configurations and thus represent a basic indicator of the mutual entanglement of a structure. Through visual inspection of configurations, as those shown in Figs 1 and 2, one verifies indeed that dimers with large |*G*′| are quite intertwined. However, from the same figures, it appears that, in addition to a global twisting of one protein around the other, *G*′ may be affected by some local details of the chains, such as their 3D shape near their ends. These local details should be irrelevant if one thermally excites the dimer, which should unfold with a time scale that corresponds to the Rouse dynamics needed to untwist one whole chain from the other^48^.

Motivated by these observations, to complement *G*′ we tackle the problem of the entanglement from a dynamical perspective. For some test dimers we monitor the evolution of the value of *G*′(*t*) with time, in a Langevin simulation where only excluded volume effects play a role (besides of course the chain connectivities). This is equivalent to the unfolding at a sufficiently high temperature.

The average curve of *G*′(*t*) over many trajectories starts from the static value of the crystallographic structure (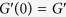) and decays to zero for long time. It turns out that an exponential form 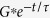 represents well the long time decay of 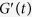. However, the extrapolated value of the fit at time *t *= 0, namely G^*^, does not necessarily match the static value *G*′. In the studied cases, shown in Fig. 7 and listed in Table 2, we find both instances of 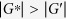 (3DIE and 1LGP), and 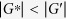 (1WKQ, 1LOM, 1M0D). This shows that G^*^, a more time consuming option than *G*′, may however be considered for complementing the quick, static evaluation of the Gaussian entanglement. Of course, any dynamical procedure provides a result that depends on the kind of dynamics used to disentangle the structure. For example, at room temperature we may expect different G^*^’s if we perform all atom molecular dynamics^49^ or Monte-Carlo methods based on local moves^50^ (where effective interactions among amino-acids are taken into account). Hence, the analysis of dimers’ intertwining through the value of *G*′ may, and should, be complemented by alternative dynamical methods, in order to get a detailed picture of the entanglement conditions.

The second parameter of the fit, the timescale *τ* (in simulation units), should increase with the chain length. This is expected for Rouse dynamics of polymers in general^48^: to shift the polymer center of mass of one radius of gyration one needs to wait a time 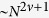 with Flory exponent *v *≈ 3/5. It is not possible for us to assess if this scaling is respected, given the few cases analysed. However, these cases correctly display an almost monotonically increasing trend of *τ* with *N* (compatibly with error bars, see Table 2). Note that strong logarithmic corrections to the scaling 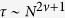 are also expected in unwinding processes^51^,^52^.

## Discussion

Mathematically, two curves are linked or not, in a rigorous sense, only if they are loops. Hence, it is not trivial to estimate the level of intertwining between two open chains. Yet, the mutual entanglement is a well-visible feature in the crystallographic structures of several domain-swapped dimers. Being interested in quantifying such entanglement, with Gauss double integrals over the backbones of the two proteins in the dimers we provide a simple and efficient indicator, the Gaussian entanglement *G*′. Indeed, according to our comparisons, a procedure for looping each protein (with an artificial continuation escaping from the core of the dimer) produces on average a linking number *G* that is strongly correlated with *G*′. This suggests that such procedure can be avoided in practice, one may just rely on the information from the open chains, encoded in *G*′.

We report that about 15% of the domain-swapped dimers have a significant 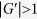. This is quite intriguing, especially if compared with corresponding figures for knotting of single proteins, where about 1% of the PDB entries has been found to host a knot^35^.

**Figure 3:**
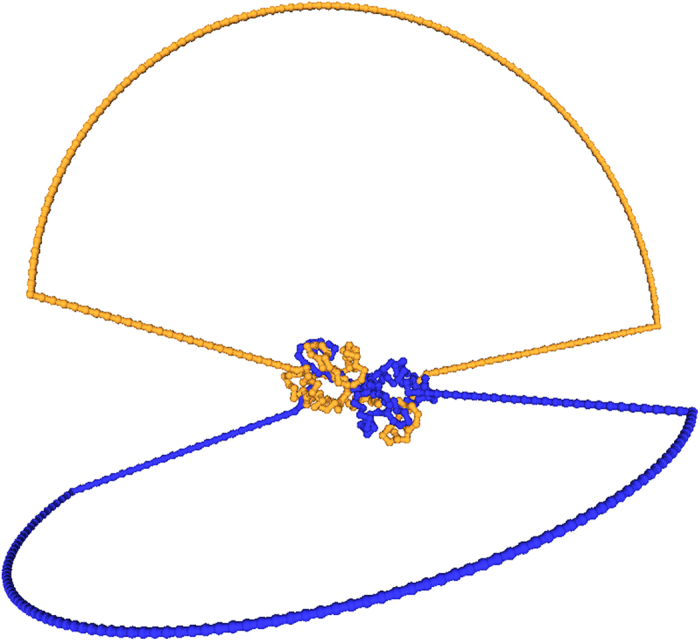
Example of closure in two loops. One of the closures of the 3DIE protein. The other closures of this dimer correspond to different orientations of the semi-circles, hinged to the straight, fixed segments.

**Figure 4:**
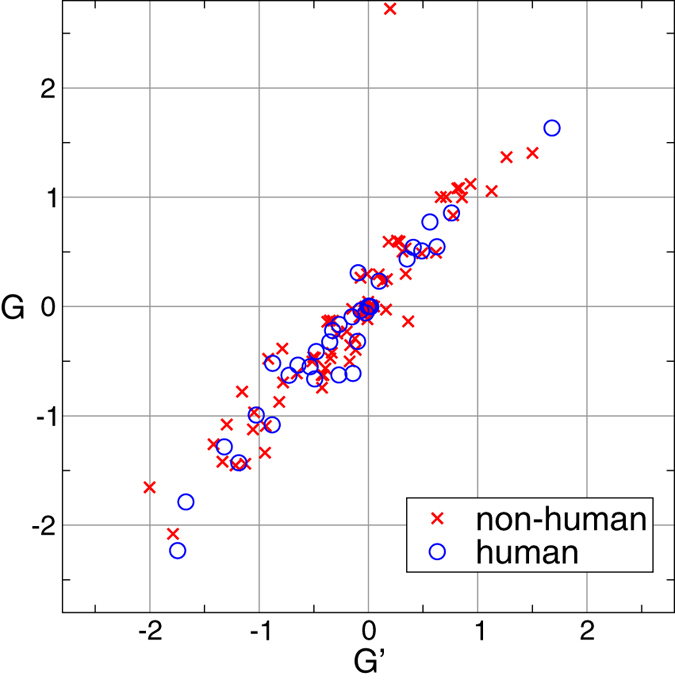
Correlation between the two measures of entanglement. Except for an outlier point, data show a good linear correlation between *G* and *G*′.

**Figure 5:**
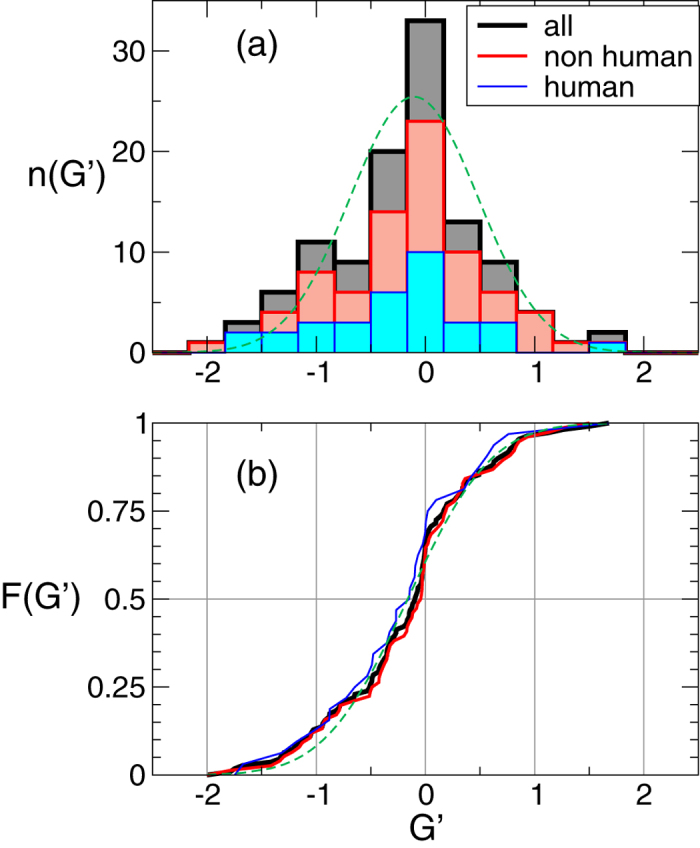
Distribution of the entanglement. (**a**) Histogram of the values of *G*′ for all swapped dimers in the database, and contributions from the groups of human and non-human dimers. The global histogram is fitted by a Gaussian distribution (dashed line) with mean *m* ≈ −0.1 and standard deviation *σ* ≈ 0.63. (**b**) Cumulative distributions of *G*′ for the same ensembles (solid lines) and an error function fit of the the global set of data (dashed line).

**Figure 6:**
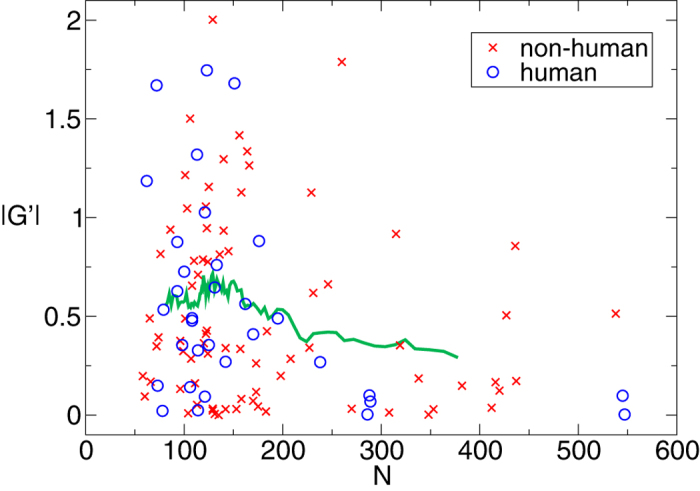
Modulus of the entanglement vs. protein length. Absolute Gaussian entanglement *G*′ as a function of the number of amino acids in one protein of a dimer. The line represents a running average over 21 points.

The asymmetry in the typical values of *G*′, with many more cases with *G*′<1 than with *G*′>1, is another interesting feature emerging from our analysis. This asymmetry could be explained by the conjecture that the Gaussian entanglement, despite being a global feature, can be deduced from the local twisting of closely interacting swapped structural elements. In several cases the latter are *β*-strands within the same sheet (see for example 1M0D, 1LGP in Fig. 1 and 3DIE, 1WKQ in Fig. 2), so that the more frequent case of a right-handed anti-parallel *β*-sheet^53^ would indeed imply a negative Gaussian entanglement. With a preliminary overview, we note that anti-parallel swapped *β*-sheets are indeed occurring more frequently in the human dimers of our database than in the non-human ones, and human dimers have indeed on average a more negative *G*′. The dependence of the intertwining of domain-swapped protein dimers on the local twist of swapped interacting elements is a feature clearly worth further investigation. If confirmed, the tuning of local interactions could then be an evolutionary mechanism used by natural selection to control the emergence of topological entanglement in domain-swapped dimers. We also tried to investigate whether there is a correlation between entanglement and pathological states. However, our analysis of domain-swapped dimers associated with pathologies^17^ does not show the emergence of any clear pattern.

The longer the proteins in the dimers, the slightly lower is their mutual entanglement. This is a surprising feature because one might anticipate that longer chains should be more easily entangled than shorter ones. Thus, it seems that the natural selection has acted against a form of random interpenetration in long domain-swapped protein dimers.

Via numerical simulations of the dissociation of some dimers, we observe that the presence of a non-trivial mutual entanglement is a robust characteristic, which vanishes exponentially with time during the dimer unbinding. The exponential fit furnishes a characteristic disentanglement time *τ* whose values does not depend on *G*′ and is weakly correlated with the length of the proteins. The amplitude of the exponential decay of the Gaussian entanglement furnishes a further estimate (G^*^) of the intertwining in the dimer, which complements the *G*′ of the crystallographic structure (they are not exactly equal to each other) in assessing the amount of linking in the dimer.

Our new approach of classifying domain-swapped protein dimers according to their mutual entanglement will likely add novel insight on the crucial role played by the generic topological properties of linear polymer chains in the protein context. As already demonstrated in the case of knotted protein folds^36^, the presence of a global topological constraint, such as the linking between two protein chains, may strongly impact the conformational properties, the thermodynamic and kinetic stability, the functional and evolutionary role of domain-swapped structures.

Finally, it is interesting to speculate on the possible outcome of a single-molecule experiment performed by pulling apart the two protein backbones of a domain-swapped dimer with significant entanglement (high |*G*′|). A similar experiment was carried on very recently for single-domain protein knots, showing that the knotting topology of the unfolded state can be controlled by varying the pulling direction^54^. In the linked dimer case, similarly, we expect the choice of the pulling directions to be crucial in allowing or not dimer unlinking and dissociation into monomers.

## Methods

### Data bank of 3D domain-swapped dimers

In order to derive a statistically significant ensemble of non-redundant domain-swapped dimers, we merge two existing databanks of domain-swapped proteins, namely *3DSwap*^55^,^56^,^57^ and *ProSwap*^58^,^59^. These databanks provide curated information and various sequence and structural features about the proteins involved in the domain swapping phenomenon. PDB entries involved in 3D Domain swapping are identified from integrated literature search and searches in PDB using advanced mining options. We first consider only the dimers and, to avoid the presence of related structures, we consider the UniprotKB code. For each code, we select only one protein, the one obtained experimentally with the highest experimental accuracy. We then filter the remaining structures to avoid the presence of holes in the main backbone chain. Specifically, we discarded those proteins in which the distance between two *C*_*α*_, listed consecutively in the pdb file, is bigger than 10 Å. As a matter of fact, such holes could affect dramatically the reliability of the linking number and there is not an obvious strategy to join them artificially. At the end we obtained a databank of *n*_*D*_ = 110 proteins, whose PDB code is reported in Table 1. Out of these, 33 were human.

### Gauss integrals

Our procedure for estimating the amount of linking between two proteins uses Gauss integrals. As representative of the backbones of proteins, we consider the chains connecting the coordinates 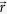 of the *C*_*α*_ atoms of amino acids, which are *N* in each of the two proteins in the dimer.

A definition of linking number between two closed curves *γ*_1_ and *γ*_2_ in 3 dimensions is given by the Gauss double integral,


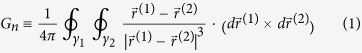


This formula yields integer numbers *G_n_* = 0, −1, 1, −2, 2, etc. Such definition is adapted to compute the amount of linking between two proteins. We need first to define a procedure that closes each open chain via the addition of artificial residues. This closure starts by computing the center of mass of the dimer and by considering such center as a repeller for the new growing arms. Let us describe the method for protein 1, as for protein 2 it is exactly the same: from each of the two free ends we continue with a path expanding diametrically from the center of mass, with a length corresponding to *n* = 25 typical *C*_*α*_ – *C*_*α*_ distances 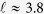 Å. At this stage the polymer is composed by *N *+ 2*n* residues. Since there is some arbitrariness in the final closure joining the two artificial new end residues, we perform a statistics over 12 different closures, each being a meridian along a sphere where the poles are the artificial end residues. Semicircular closure paths are drawn, each containing a number of artificial residues *n*′ that makes their bond distance as close as possible to 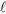. An example is shown in Fig. 3.

Each closed chain becomes a collection of 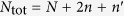 points 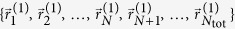 separated by about a fixed spacing 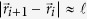, so that the integrals are replaced by sums over segments 
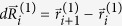
, for which we use the midpoint approximation 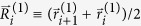. For a given choice *z* of the closure for both proteins, out of the 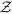 = 12 × 12=144 possible ones, we have


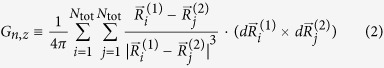


(indices run periodically, hence 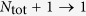, and the notation leaves the dependence of 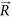
’s on *z* understood), and the final estimate of linking is an average over *Z* closures


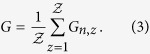


A closure provides an integer linking number *G*_*n,z*_, though the final average *G* may become not integer if closures with different *G*_*n,z*_ are generated. As an alternative, we relax the requirement to have basic integer indicators of linking and we perform the double Gauss discrete integral over the open chains. The Gaussian entanglement indicator


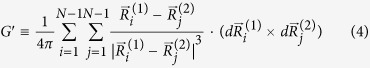


has no statistical averaging and is a straightforward alternative to *G* in the estimate of the linking of proteins.

### Simulations

In our molecular dynamics simulations, each protein forming the dimer is modeled as a self-avoiding chain of *N* beads. Each bead has radius *σ* and is centered in the *C*_*α*_ position of a residue. Adjacent beads of each protein are tethered together into a polymer chain by an harmonic potential with the average *C*_*α*_ − *C*_*α*_ distance along the chain equal to 1.5 *σ*. To take into account the excluded volume interaction between beads we consider the truncated Lennard-Jones potential


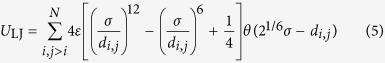


where 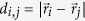 is the distance of the bead centers *i* and *j*, *θ* is the Heaviside function and *ε* is the characteristic unit of energy of the system which is set equal to the thermal energy *k*_*B*_*T*.

The system dynamics is described within a Langevin scheme:


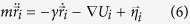


where *U*_1_ is the total potential of the *i*^th^ particle, *γ* is the friction coefficient and *η* is the stochastic delta-correlated noise. The variance of each Cartesian component of the noise, 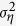 satisfies the usual fluctuation dissipation relationship 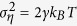. As customary, we set 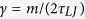, with 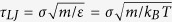 being the characteristic simulation time. From the Stokes friction coefficient of spherical beads of diameters *σ* we have 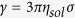, where *η* is the solution viscosity. By using the nominal water viscosity, 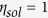 cP and setting *T* = 300 K and *σ* = 2.5 nm, one has 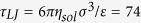 ns.

**Figure 7:**
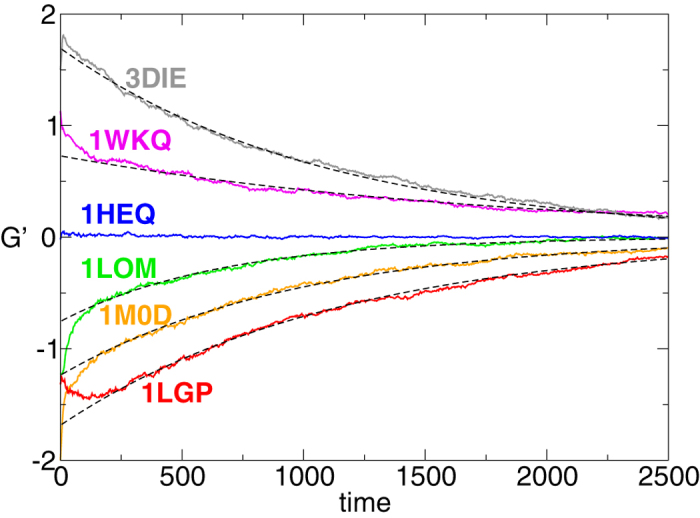
Decay of the entanglement with time, during unfolding. Time dependence of *G*′ during the unfolding dynamics of a set of swapped dimers. The dashed curves correspond to the best fit of the data with the function 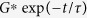. Note that, to better catch the exponential decay, the first 100 time steps have been neglected in the fit.

To study the unfolding dynamics of a given dimer, we take its folded configuration, as given by the PDB, as the initial condition. For each initial condition we generate 100 different molecular dynamics trajectories by integrating numerically (6) up to 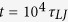. During the dynamics we monitor the quantities *G* and *G*′. In Fig. 7 the curves are obtained by averaging over the 100 trajectories. Simulations are performed with the package LAMMPS^60^.

## Additional Information

**How to cite this article**: Baiesi, M. *et al*. Linking in domain-swapped protein dimers. *Sci. Rep*. **6**, 33872; doi: 10.1038/srep33872 (2016).

## Figures and Tables

**Table 1:** Domain-swapped dimers ranked from lowest to highest Gaussian entanglement *G*′.

dimer	*G*′	*G*	*N*		dimer	*G*′	*G*	*N*		dimer	*G*′	*G*	*N*	
1M0D	−2	−1.65	129		1K51	−0.35	−0.47	72		1BJ3	0.02	0	129	
2J6G	−1.79	−2.08	260		2A62	−0.35	−0.13	319		2QYP	0.02	0	78	h
2XDP	−1.75	−2.23	123	h	1N9J	−0.35	−0.32	98	h	1 × 2W	0.03	0.01	129	
2Z0W	−1.67	−1.79	72	h	1K4Z	−0.33	−0.42	157		1QB3	0.05	0	113	
1I1D	−1.42	−1.26	156		1TIJ	−0.33	−0.22	114	h	1AOJ	0.09	0.29	60	
2P1J	−1.34	−1.42	164		1ZVN	−0.32	−0.13	99		1S8O	0.1	0.23	545	h
1LGP	−1.32	−1.28	113	h	2A4E	−0.28	−0.25	208		1CDC	0.13	0.23	96	
1NPB	−1.3	−1.08	140		1DXX	−0.27	−0.17	238	h	1WY9	0.16	−0.03	111	
1LOM	−1.22	−1.45	101		1K04	−0.27	−0.63	142	h	3NG2	0.17	0.25	66	
2BZY	−1.19	−1.43	62	h	3FJ5	−0.2	−0.23	58		2HZL	0.19	0.59	338	
1KLL	−1.16	−0.78	125		1CTS	−0.17	−0.5	437		2FPN	0.2	2.72	198	
1HW7	−1.13	−1.44	229		1FOL	−0.17	−0.35	416		2CN4	0.26	0.6	173	
1BYL	−1.06	−1.12	122		2BI4	−0.15	−0.02	382		2SPC	0.29	0.6	107	
1MI7	−1.05	−0.97	103		2ONT	−0.15	−0.09	73	h	1R5C	0.31	0.5	124	
1BUO	−1.03	−0.99	121	h	2CI8	−0.14	−0.61	106	h	2CO3	0.34	0.53	142	
1O4W	−0.95	−1.34	123		1DWW	−0.12	−0.29	420		2OQR	0.34	0.3	227	
1MU4	−0.94	−1.09	86		1QQ2	−0.12	−0.4	173		1H8X	0.35	0.44	125	h
1W5F	−0.92	−0.48	315		1XMM	−0.1	−0.32	288	h	3HXS	0.36	−0.14	120	
1FRO	−0.88	−1.08	176	h	1Q8M	−0.09	0.31	121	h	1GP9	0.41	0.54	170	h
2VAJ	−0.88	−0.52	93	h	1GT1	−0.08	−0.1	158		2FQM	0.49	0.49	65	
1HT9	−0.82	−0.87	76		1NNQ	−0.07	0.26	170		2DSC	0.49	0.51	195	h
3FSV	−0.79	−0.38	119		3D94	−0.07	−0.04	289	h	1SK4	0.56	0.77	162	h
1ZK9	−0.78	−0.69	110		2IV0	−0.04	−0.04	412		2DI3	0.62	0.49	231	
3LOW	−0.73	−0.63	100	h	2W1T	−0.04	−0.07	175		2NZ7	0.63	0.55	93	h
1T92	−0.66	−0.61	108		1L5X	−0.03	−0.04	270		2HN1	0.66	1	246	
2ES0	−0.65	−0.54	131	h	2E6U	−0.03	−0.02	142		1OSY	0.71	1	114	
2RCZ	−0.53	−0.55	79	h	2O7M	−0.03	−0.04	153		2A9U	0.76	0.86	133	h
1KAE	−0.51	−0.46	427		4AEO	−0.03	0	353		1A2W	0.78	0.83	124	
1NIR	−0.51	−0.5	538		3PSN	−0.02	−0.03	183		1QX7	0.81	1.08	136	
1PUC	−0.49	−0.47	101		1HE7	−0.02	−0.06	114	h	1QX5	0.83	1.09	145	
1HUL	−0.49	−0.66	108	h	1U4N	−0.01	0.3	308		1MV8	0.86	1	436	
1I4M	−0.48	−0.41	108	h	1UKM	−0.01	−0.01	131		1QWI	0.93	1.12	140	
2NU5	−0.43	−0.63	123		1YGT	−0.01	−0.12	104		1WKQ	1.13	1.06	158	
3LYQ	−0.42	−0.74	184		1XUZ	0	0.04	348		1E7L	1.26	1.37	166	
2GUD	−0.41	−0.63	122		2PA7	0	−0.04	135		3DIE	1.5	1.41	106	
1R7H	−0.39	−0.57	74		1VJ5	0	0	547	h	1ILK	1.68	1.63	151	h
1ZXK	−0.38	−0.14	96		2JFL	0	0	286	h					

The mean linking number (*G*) and the number of amino acids in each protein of the dimer (*N*) are also reported for the analysed dimers. Human proteins are tagged with a “h”.

**Table 2:** Relaxation time *τ* and entanglement indicators, for some swapped dimers.

dimer	*N*	*τ*	G^*^	*G*′	*G*
1M0D	129	1000 (70)	−125	−2.00	−1.65
1LGP	113	1140 (80)	−1.70	−1.33	−1.28
1LOM	101	660 (80)	−0.77	−1.23	−1.46
1WKQ	155	2500 (120)	0.72	1.13	1.07
3DIE	107	1090 (90)	1.68	1.50	1.41
